# Enhanced dielectricity coupled to spin-crossover in a one-dimensional polymer iron(ii) incorporating tetrathiafulvalene†
†Electronic supplementary information (ESI) available. CCDC 1835730, 1835731 and 1942248. For ESI and crystallographic data in CIF or other electronic format see DOI: 10.1039/d0sc02388d


**DOI:** 10.1039/d0sc02388d

**Published:** 2020-05-27

**Authors:** Ya-Ru Qiu, Long Cui, Pei-Yu Cai, Fei Yu, Mohamedally Kurmoo, Chanel F. Leong, Deanna M. D'Alessandro, Jing-Lin Zuo

**Affiliations:** a State Key Laboratory of Coordination Chemistry , School of Chemistry and Chemical Engineering , Collaborative Innovation Center of Advanced Microstructures , Nanjing University , Nanjing 210023 , P. R. China . Email: zuojl@nju.edu.cn; b School of Chemistry and Materials Science , Nanjing University of Information Science and Technology , Nanjing , 210044 , P. R. China; c Institut de Chimie de Strasbourg , CNRS-UMR7177 , Université de Strasbourg , 4 rue Blaise Pascal , Strasbourg 67000 , France . Email: kurmoo@nju.edu.cn; d School of Chemistry , The University of Sydney , Sydney , New South Wales 2006 , Australia

## Abstract

In designing multifunctional materials for potential switches that can be used as memory devices, the high-spin (HS) to low-spin (LS) crossover (SCO) one-dimensional polymer, [Fe^II^(L)(4,4′-bpy)]_*n*_, was constructed from a designed redox-active tetrathiafulvalene (TTF) functionalized Schiff-base and the ditopic linker 4,4′-bipyridine (bpy). It exhibits an 8 K hysteretic SCO centred at *T*_1/2_ = 325 K which is coupled to changes in its dielectric constant. The crystal structures above and below the transition temperature reveal similar parallel linear ···Fe–bpy–Fe–bpy··· chains displaying expansion of the Fe^II^ octahedron in the HS state. Density functional theory (DFT) calculations reveal a concerted electronic charge and spin change represented by the Mülliken charge of the Fe and the magnitude and direction of the dipole moment which substantiate the experimental observations.

## Abstract 2

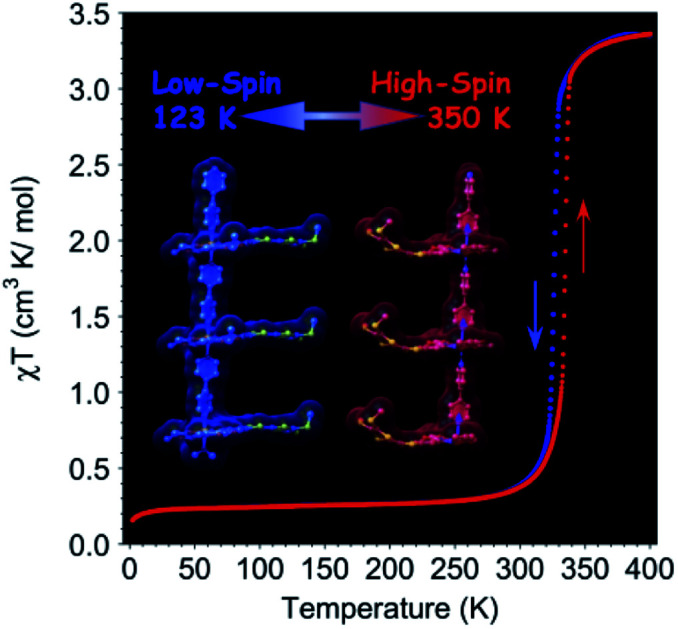
A concerted bending–flattening motion of the redox-active TTF within constructed one-dimensional Fe^II^–TTF–Schiff-base chain with bridging 4,4′-bpy enhances the dielectric constant coupled to its spin-crossover transition above room temperature.

## Introduction

Since the first observation of spin-crossover (SCO) in an Fe^II^ compound was reported in 1964,^1^ the phenomenon has fascinated both physicists and chemists alike. While its origin was readily identified, the effects of SCO on changes in volume, entropy, dielectricity and colour have driven an extensive line of research in view of possible fabrication of memory devices for applications. As the physical properties are being explained theoretically, chemists have been producing endless variations of materials with designable structures of all dimensionalities. In addition, other physical and chemical properties have been incorporated in view of developing multifunctional intelligent materials.^2^ Up to now, a large number of complexes have been prepared from metal ions with d^4^–d^7^ electronic configurations using a variety of ligands.^3^ The most notable systems are those based on metals with the d^6^ configuration, such as Fe^II^, that exhibit bi-stability around room temperature, where the transitions can be tuned by various external physical perturbations; these include temperature, pressure, light or X-ray irradiation, and intense magnetic fields, as well as chemical stimuli such as a change in the guest molecules or by their removal or inclusion.2c–f,^4^ Significant research efforts are focused on widening the hysteresis width around room temperature.2b,^5^ From a chemistry perspective, the development of Fe^II^ based SCO has evolved from complexes containing solely nitrogen donor ligands such as pyridine due to its moderate ligand field, to those containing both nitrogen and oxygen donors, such as Schiff-base ligands. In this context, Kou *et al.* achieved multifunctional SCO in two Fe^II^–Schiff-base complexes which displayed SCO at *ca.* 300 K, which was controllable by dehydration-hydration.^6^ Recently, Weber *et al.* investigated a one-dimensional (1D) SCO polymer [Fe^II^(L_1_)(4,4′-bpy)]_*n*_ (L_1_ is a N_2_O_2_^2–^ coordinating Schiff-base bearing a phenazine fluorophore) possessing a 48 K wide hysteresis above 300 K, where the SCO could also be monitored by the ligand luminescence.^7^

Thus, the idea of functionalizing a Schiff-base ligand with a probe segment is an effective strategy to develop switchable SCO materials. In this regard, a new approach is the development of a system incorporating a redox-active pendant to control the Fe^II^ SCO transition. Tetrathiafulvalene (TTF), a sulphur-rich conjugated molecule possessing two reversible and easily accessible oxidation states (*i.e.*, radical TTF˙^+^ and diamagnetic TTF^2+^), represents a potential molecule that can be appended to a Schiff-base. TTF is well known as an appealing electron donor, and exhibits rich redox chemistry that underpins its application in studies of electrochromic materials, electrocatalysis and photoconductive switches.3f,^8^ Furthermore, complexes bearing TTF are highly capable of stacking into supramolecular structures and facilitating efficient pathways for charge transport due to π···π and S···S interactions.^9^

**Scheme 1:**
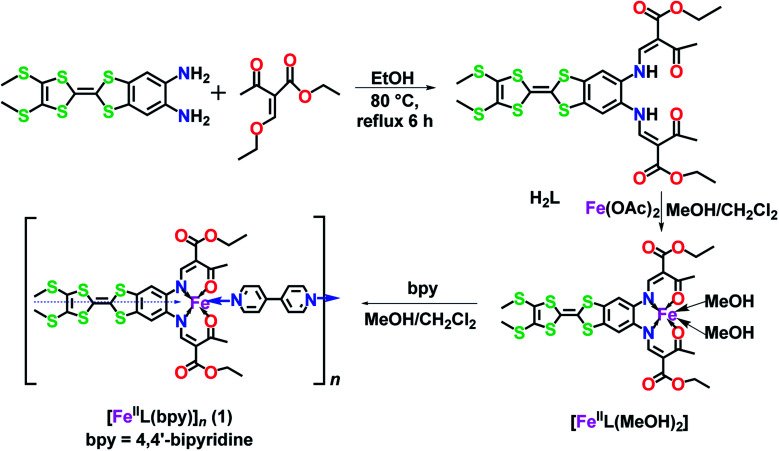
Design approach for a TTF–Schiff-base ligand (**H_2_L**) and its iron(ii) precursor [Fe^II^(L)(CH_3_OH)_2_] used in the formation of the 1D-spin-crossover polymer [Fe^II^(L)bpy]_*n*_.

In view of the fascinating structures that have been reported for TTF functionalized ligands, such as TTF–(1,2,3-triazole), tetra(4-pyridyl)–TTF, and tetra(4-benzoate)–TTF, in coordination compounds,3f,8c,9b,^10^ we have appended TTF to a Schiff-base. By adding the redox activity of the TTF to the N_2_O_2_ square planar unit of the Schiff-base, we propose the development of a new class of multifunctional SCO materials (Scheme 1). In this context, only one mononuclear Fe^II^ ligated to a tetrathiafulvalene-functionalized dipyridophenazine has been reported to date.^11^ Both thermal spin-crossover (*ca.* 143 K) and a wide hysteresis of 48 K were observed. Cyclic voltammetry in solution identified the two oxidation steps of the TTF. However, in the absence of structural analyses of the low-spin state and dielectric measurements, no further insights were gleaned into the coexisting redox and SCO properties. Herein, we report a new Fe^II^ SCO 1D-polymer, [Fe^II^(L)(bpy)]_*n*_ (**1**), with an additional bridging 4,4′-bpy. This new material is found to be an exemplary compound displaying a concerted tandem cooperation between the SCO and dielectricity. While the dielectric anomaly is often explained by the contraction of the Fe octahedron from high-spin (HS) to low-spin (LS), we propose a further reason for enhancing the effect which is driven by bending of the TTF moiety, altering the magnitude and direction of the dipole moment.

## Experimental section

The methods used for elemental analyses, thermogravimetry, infrared spectroscopy, single crystal X-ray diffraction data collection and the structural analyses, magnetic, optical and electrical property determinations as well as the DFT calculations are detailed in the ESI† to avoid repetition from our previous works. The preparation of [Fe^II^L(bpy)] was realized in three steps as follows from **H_2_L**, [Fe^II^L(MeOH)_2_], and [Fe^II^L(bpy)].

### Synthesis of ligand **H_2_L**

2-(4,5-Bis(methylthio)-1,3-dithiol-2-ylidene)benzo[*d*][1,3]dithiole-5,6-diamine (376 mg, 1 mmol) and ethyl 2-acetyl-3-ethoxyacrylate (559 mg, 3 mmol) were dissolved in 15 mL ethanol and heated at reflux for 6 h. After cooling to 25 °C, **H_2_L** (orange-yellow solid) was collected by filtration. Yield: 550 mg (84%). Anal. calc. for C_26_H_28_N_2_O_6_S_6_: C 47.54, H 4.30, N 4.26, S 29.29%; found: C 47.33, H 4.15, N 4.06, S 29.15%. ^1^H NMR (400 MHz, CDCl_3_, ppm): *δ* 12.95 (d, *J* = 12.1 Hz, 1H), 8.27 (d, *J* = 12.1 Hz, 2H), 7.13 (s, 1H), 4.27 (q, *J* = 7.1 Hz, 4H), 2.57 (s, 3H), 2.45 (s, 3H), 1.34 (t, *J* = 7.1 Hz, 6H). IR (KBr pellet, cm^–1^): 2980 (w), 1700 (s), 1635 (s), 1580 (s), 1404 (m), 1254 (s), 1195 (m), 1066 (s), 774 (m).

### Synthesis of [Fe^II^L(MeOH)_2_]

**H_2_L** (132 mg, 0.2 mmol) and anhydrous ferrous acetate (42 mg, 0.24 mmol) were dissolved in 5 mL CH_2_Cl_2_ and 15 mL MeOH in a N_2_ glove box. After stirring overnight at 25 °C, the resulting dark green solid of [Fe^II^L(MeOH)_2_] was collected by filtration. Yield: 100 mg (65%, based on **H_2_L**). Anal. calc. for C_28_H_34_FeN_2_O_8_S_6_: C 43.40, H 4.42, N 3.62, S 24.83%; found: C 43.16, H 4.18, N 3.49, S 24.53%. IR (KBr pellet, cm^–1^): 2975 (w), 1711 (s), 1628 (s), 1589 (m), 1427 (s), 1379 (m), 1261 (s), 1073 (s), 849 (w), 774 (w).

### Synthesis of [Fe^II^L(bpy)]_*n*_ (**1**)

[Fe^II^L(MeOH)_2_] (50 mg, 65 μmol) and bpy (11 mg, 70 μmol) were dissolved in 15 mL CH_2_Cl_2_ and 5 mL MeOH in a glove box. After stirring overnight at room temperature, the mixture was filtered and black needle crystals of [Fe^II^L(bpy)] were collected after partial vaporization of the solvents. Yield: 38 mg (68%, based on [Fe^II^L(MeOH)_2_]). Anal. calc. for C_36_H_34_FeN_4_O_6_S_6_: C 49.88, H 3.95, N 6.46, S 22.19%; found: C 49.63, H 3.75, N 6.29, S 22.01%. IR (KBr pellet, cm^–1^): 2921 (w), 1684 (s), 1587 (s), 1427 (s), 1261 (s), 1197 (w), 1073 (m), 852 (w), 771 (w).

## Results and discussion

Due to the very rapid loss of solvent of crystallization all reported measurements (including magnetic susceptibility, differential scanning calorimetry, dielectric permittivity, solid-state cyclic voltammetry, and electrical conductivity), except that for single-crystal diffraction at 123 K, were performed on fully desolvated samples.

**Fig. 1:**
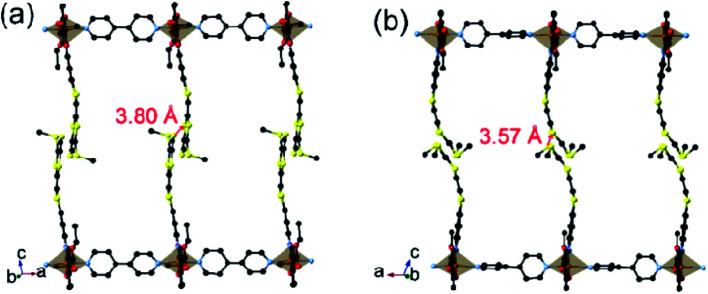
Views of a pair of supramolecular chains in the structures of **1** in its (a) LS state at 123 K and (b) HS state at 350 K. The S···S contacts between neighbouring TTF moieties are shown as red colour sticks.

At 123 K, the triclinic *P*-1 unit-cell of **1** contains one Fe, one TTF–Schiff-base and one bpy (Table 1). The Fe^II^ (Fe1) adopts a distorted octahedron where two nitrogen atoms (N1 and N2) and two oxygen atoms (O1 and O2) from the TTF–Schiff-base form the basal plane, and two nitrogen atoms from the bridging bpy (N3 and N4) occupy the apical positions. The sum of angles between the basal atoms is 360°, suggesting that the atoms Fe1, O1, O2, N1, and N2 share the same plane. The axial N3–Fe1–N4 angle of 175.4° indicates that the Fe1, N3, and N4 are nearly in a line. Each bpy bridges two Fe^II^ to construct linear 1D ···Fe–bpy–Fe–bpy··· chains, and short S···S contacts between the TTF moieties of adjacent parallel chains, leading to ladders (Fig. 1 and S1†). Intermolecular interactions (C2···O1) result in a two-dimensional (2D) network. The average Fe1–N and Fe1–N_bpy_ bond lengths are 1.982 and 2.100 Å, respectively, and lie within the range reported for low-spin Fe^II^ (Fe1) compounds.3c,^12^

**Table 1:** Crystallographic and structure refinement data for **1** and **H_2_L**

Compounds	**1**-123 K	**1**-350 K	**H_2_L**
Empirical formula	C_36_H_34_FeN_4_O_6_S_6_·CH_2_Cl_2_	C_36_H_34_FeN_4_O_6_S_6_	C_28_H_31_N_3_O_6_S_6_
*M*_r_	951.81	866.88	697.92
*T* [K]	123	350	296
*λ* [Å]	0.71073	0.71073	0.71073
Crystal system	Triclinic	Triclinic	Monoclinic
Space group	*P*1[combining macron]	*P*1[combining macron]	*P*2_1_/*c*
*a* [Å]	11.3346(16)	11.360(11)	4.7950(15)
*b* [Å]	12.2013(17)	12.085(10)	25.807(8)
*c* [Å]	18.242(3)	16.842(14)	26.902(8)
*α* [°]	98.812(5)	91.22(2)	90
*β* [°]	99.395(5)	109.72(2)	91.827(6)
*γ* [°]	113.676(3)	111.47(2)	90
*V* [Å^3^]	2211.6(5)	1998(3)	3327.2(18)
*Z*	2	2	4
*d*_calc_ (g cm^–3^)	1.302	1.441	1.393
*μ* (mm^–1^)	0.67	0.74	0.46
*F*(000)	896	896	1456
Refl. total	13 771	12 227	22 458
Refl. unique	7579	6358	18 416
GOF (*F*^2^)	1.04	1.09	1.03
*R*_1_ ^*a*^, w*R*_2_^*b*^ [*I* > 2*σ*(*I*)]	0.0840, 0.1861	0.1290, 0.2426	0.0621, 0.1391
*R*_1_ ^*a*^, w*R*_2_^*b*^ [all data]	0.1012, 0.1969	0.2822, 0.3013	0.1152, 0.1608

^*a*^ *R*_1_ = ∑‖*F*_o_| – |*F*_c_‖/∑|*F*_o_|.

^*b*^w*R*_2_ = [∑_*w*_(*F*_o_^2^ – *F*_c_^2^)^2^/∑_*w*_(*F*_o_^2^)^2^]^1/2^.

Upon heating to 350 K, the key structural features measured at 123 K remain, but the Fe^II^ (Fe1) forms longer bonds (average Fe1–N of 2.079 Å, Fe1–N_bpy_ of 2.197 Å, and Fe1–O of 1.994 Å), characteristic of reported compounds in the HS state.^7^,^12^,^13^ The in-chain Fe···Fe separations are 11.34 Å (123 K) and 11.36 Å (350 K). In addition to the expansion of the Fe^II^ (Fe1) coordination sphere, the angles in the LS state tend to increase upon changing to HS. The angles O1–Fe1–O2 = 99.1° and average N1–Fe1–N2 = 82.2° at 123 K, become 110.1° and 79.7° at 350 K. Selected bond lengths and angles are listed in Table S1.†

**Fig. 2:**
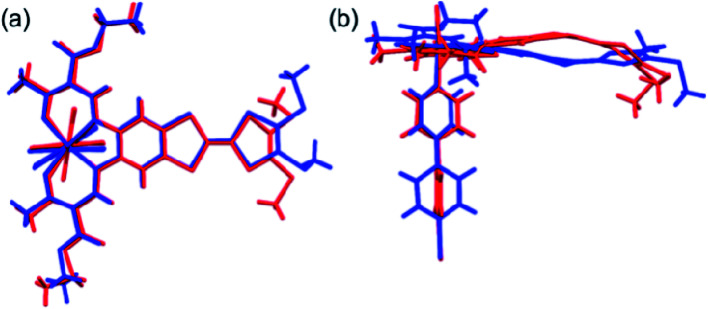
Top (a) and side (b) views of the superposition of the basic molecular skeletons of the structures at 123 K (blue) and 350 K (red).

The most striking difference between the LS and HS structures is the change of the TTF from planarity at 123 K to a puckered shape at 350 K (Table 2). The dihedral angle between the two pseudo-planar 5-membered rings about the central C=C bond is 18.2° (LS) and 34.7° (HS), respectively. These changes suggest that thermal vibrations may cause configuration distortion. The severe distortion is accompanied by a considerable change in orientation as demonstrated by the S···S contacts of 3.80 Å at 123 K (LS state) to 3.57 Å at 350 K (HS state). By centring the Fe and aligning the Fe–N_bpy_ bond in a superposition picture of the two structures (Fig. 2), several major structural changes in the pendant fragments of the Schiff-base are evident, as well as the rotation of the pyridine of the bpy.

**Table 2:** The comparison of C–C and C–S bond distances (Å) of the central TTF moiety for **1** and **H_2_L**

	**1**-123 K	**1**-350 K	**H_2_L**
C21–C22	1.350(3)	1.350(17)	1.335(6)
S1–C21	1.763(10)	1.721(14)	1.761(4)
S1–C18	1.750(4)	1.703(12)	1.754(4)
S2–C21	1.770(2)	1.709(14)	1.763(5)
S2–C17	1.754(10)	1.699(12)	1.754(4)
S3–C22	1.770(2)	1.719(15)	1.758(5)
S3–C24	1.750(2)	1.704(18)	1.760(5)
S4–C22	1.790(4)	1.740(15)	1.751(4)
S4–C23	1.751(10)	1.710(2)	1.763(5)

**Fig. 3:**
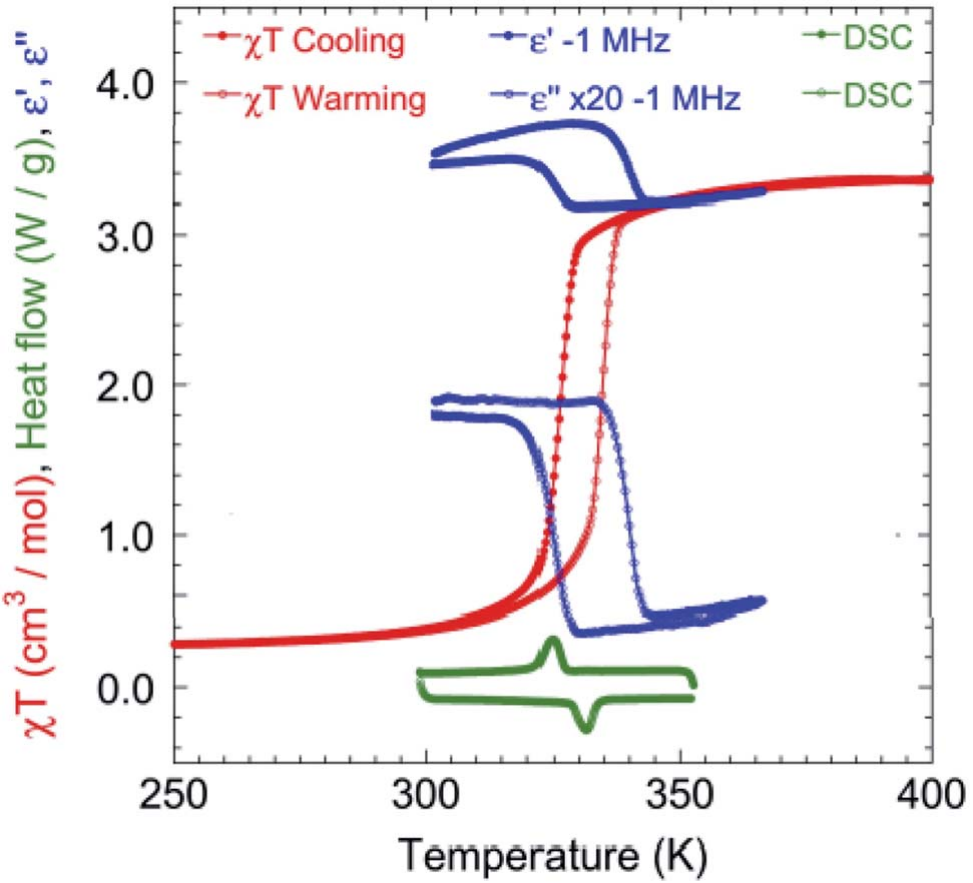
Temperature dependence of *χ*_M_*Τ* in 1 kOe (red) at a rate of 2 K min^–1^ (see Fig. S2† for the full temperature range of *χ*_M_*Τ*), real and imaginary (×20) dielectricity (blue) at a rate of 20 K min^–1^ and DSC (green) at a rate of 15 K min^–1^ around the HS–LS transition.

The magnetic susceptibility was measured between 2 and 400 K on cooling and warming in a 1 kOe field using a SQUID magnetometer (Fig. S2†). The *χ*_M_*Τ* value is 3.35 cm^3^ K mol^–1^ at 400 K, which is consistent with that expected for HS Fe^II^.^7^,12c On cooling, *χ*_M_*Τ* decreases slowly to 2.90 cm^3^ K mol^–1^ until 330 K. Upon further lowering the temperature, a sharp drop to 0.48 cm^3^ K mol^–1^ at 310 K is observed, indicating that a major fraction of the Fe^II^ centres are now in a LS state.^7^,12c Below 300 K, *χ*_M_*Τ* is almost constant at 0.24 cm^3^ K mol^–1^. Upon warming, it follows a similar trend but with a hysteresis of 8.1 K around the SCO transition (326.6 K for cooling and 334.7 K for warming, Fig. 3). At 300 and 90 K the EPR spectra are silent eliminating the possibility of the formation of a radical TTF (Fig. S3†).

Differential Scanning Calorimetry (DSC) measurements (Fig. 3 and S4†) were performed at different cooling and warming rates of 15, 10 and 5 K min^–1^. The results show an endothermic change at 331.3 K on cooling and an exothermic one at 324.9 K on warming, corresponding to the SCO transition and matching the magnetization data. The integrated enthalpy and entropy values are Δ*H* = 6.78 kJ mol^–1^ and Δ*S* = 20.9 J mol^–1^ K^–1^ for the HS to LS transition, and Δ*H* = 6.07 kJ mol^–1^ and Δ*S* = 18.3 J mol^–1^ K^–1^ for the LS to HS transition, which are consistent with those reported in the literature.3c,12c,^14^

The variation of the complex dielectric permittivity (*ε* = *ε*′ – i*ε*′′) was measured on two different samples by cycling the temperature (20 K min^–1^) from 290 to 363 K at different frequencies of 0.5, 1, 5, 10, 100, and 1000 kHz (Fig. 3 and S5†). There were anomalies discernible. Between 310 and 350 K there is a hysteresis accompanying the SCO transition. While the value of the real part (*ε*′) is low at 350 K and high at 310 K for all frequencies, that of the imaginary part goes from high to low at 500 Hz and gradually reverses from low to high between 1 and 5 kHz. The hysteresis width is independent of the frequency. The hysteresis in the real part goes from high in the HS state to low in the LS state. The maximum value of *ε*′ is *ca.* 14 for the frequency of 500 Hz, and this dielectric constant value is higher than that for most of the investigated SCO materials (Table S2†).14c,^15^ Both the reversed hysteresis and the enhancement are in contrast to what have been observed previously for compounds without the redox-active ligand. The presence of these two anomalies are considered to be driven by two concomitant effects, namely, the size change and bending of TTF.

The transition temperatures (*T*_1/2_↓ and *T*_1/2_↑), as well as the width of the thermal hysteresis (Δ*T*), and the change in *ε*′ during the SCO transition (Δ*ε*′ = *ε*′_HS_ – *ε*′_LS_) are also in agreement with the DSC and magnetization data (Fig. 3).^16^ Upon HS ↔ LS spin crossover, the variation in dielectric constant originates from the size of the breathing of the Fe octahedron and in the present case, additionally to the bending of the TTF during the SCO transition.^17^,^18^

The electrochemical properties of **1** as a solid material were investigated to gain insight into the prospects for redox modulation of the components. Cyclic voltammetry (CV) measurements in 0.1 M [(*n*-Bu)_4_N]PF_6_ in CH_3_CN supporting electrolyte (Fig. S6†) revealed a 1e^–^ oxidation for TTF to TTF˙^+^ at 0.11 V followed by a multi-electron wave at ∼0.5 V. The latter occurs at a voltage consistent with oxidation of TTF˙^+^ to TTF^2+^.^11^,^19^ At more anodic potentials, multiple irreversible processes were observed in the CV; the origin of these processes is tentatively ascribed to oxidation of the organic moieties of L. A contribution from the Fe^II/III^ couple was discounted, as a related Schiff-base iron ligand has previously been shown to have a quasi-reversible Fe^II/III^ couple at potentials below –1 V (*vs.* Fc/Fc^+^).^20^ As the potential was swept back to 0 V, the reverse processes were suggestive of reduction, however they were clearly irreversible. The second cycle of the CV experiment showed only two reversible 1e^–^ oxidation steps for the TTF to its TTF˙^+^ and TTF^2+^ forms. These observations are tentatively attributed to decomposition and dissolution of the material at a voltage above 0.5 V, such that only solution-based processes for the electrochemically active TTF constituents are observed. Indeed, subsequent runs show progressive diminishing intensity of the waves until the CV becomes largely featureless.

The electrical conductivity was measured between 300 and 350 K on warming in a Quantum Design SQUID physical property measurement system (PPMS) at the rate of 1 K min^–1^ (Fig. S7†). The electrical conductivity of **1** is 1.42 × 10^–8^ S cm^–1^ at 300 K and decreases gradually upon warming to a minimum of 8.07 × 10^–9^ S cm^–1^ at 327 K. Upon further warming, it increases over two orders of magnitude to 3.39 × 10^–7^ S cm^–1^ at 350 K. Indeed, an anomaly in the conductivity is observed around 330 K, which is close to the SCO found in the magnetic susceptibility data (Fig. S7†). In the absence of infinite stacking of the TTF in the structure, and the presence of integral charges without any mixed valency of the TTF or Fe, the crystals are insulating as observed. Thus, although the crystals are expected to be wide-band gap semiconductors, they show a maximum in electrical conductivity coinciding with the SCO transition.

Electric polarization arises from the polar displacement of anions and cations, lattice distortions, or metal-to-metal charge transfer (MMCT) and metal-to-ligand charge transfer (MLCT).^21^ Intramolecular charge transfer induces a significant change in the electronic transition within the molecule and in the magnetic properties.^22^–^24^ Therefore, charge transfer involving a change in polarization can be clearly detected by changes in the dielectric properties and magnetization measurements. To understand the origin of the dielectric anomalies, we performed DFT calculations on two phases (Table 3). At the b3lyp/6-31G** level of theory, the calculated spin densities of the Fe^II^ centres are 3.66 for the quintet spin state and 0.00 for the singlet spin state (Table 3). The range of spin densities induced a change in the electric dipole moment from 4.37 D for the quintet spin state to 9.51 D for the singlet spin state, suggesting polarity conversion during thermally induced charge transfer. The results are quite clear about an SCO transition with spin-densities of 3.7 at 350 K and 0 at 123 K. In addition, the relative reduction of Mülliken charge on the Fe centre of 25% substantiates some form of reorganisation. The increase of 19% in the dipole moment from HS to LS and the change in vector direction are the likely causes for the dielectric enhancement observed.

**Table 3:** Energies, electric dipole moments, dipole moment vectors, spin densities, Mülliken charges at the b3lyp/6-31G**levels for **1**

	Spin state	Free energy (eV)	Dipole moment (Debye)	Dipole moment vector	Spin density (Fe)	Mülliken charges (Fe)
b3lyp/6-31G**	High spin (quintet)	–12 449, 2.07	4.37	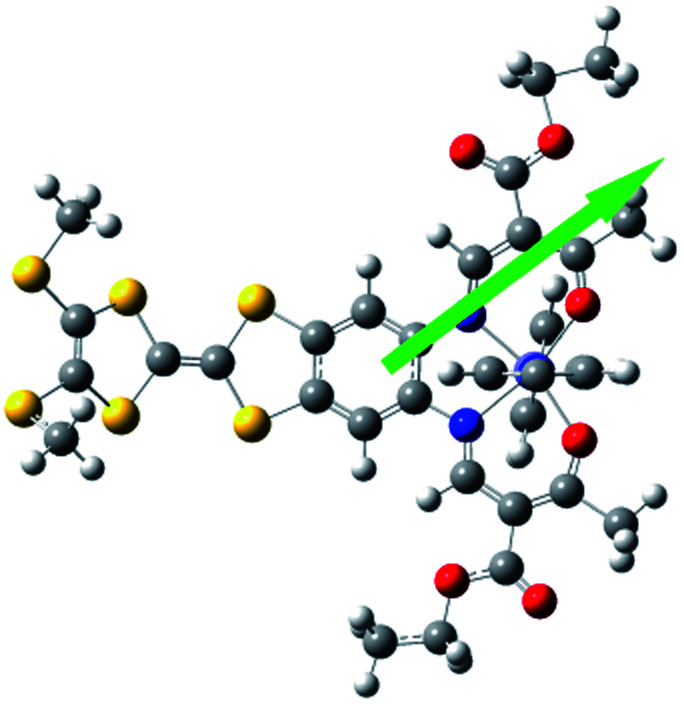	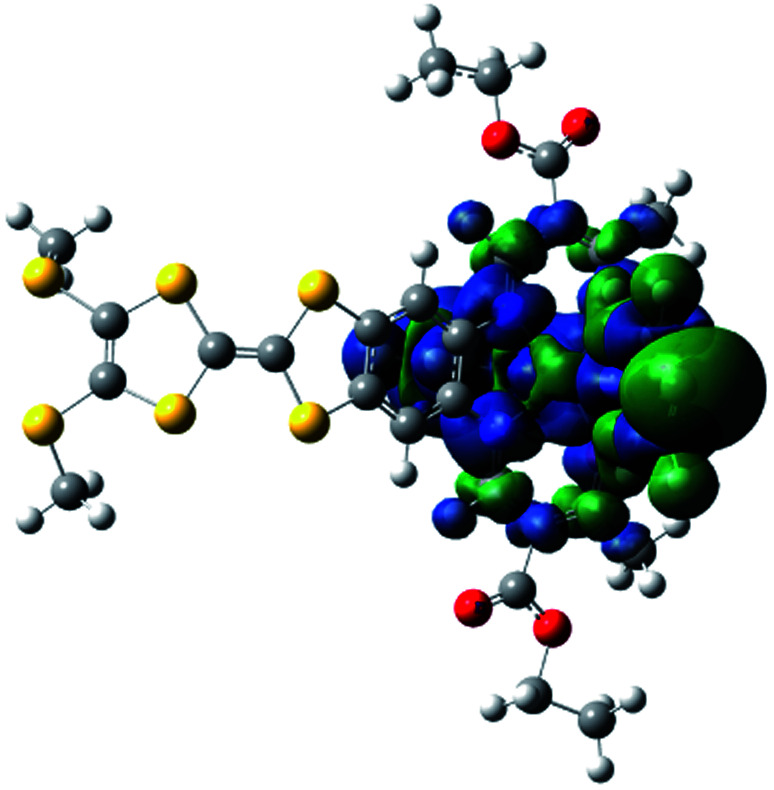 (3.66)	0.68
	Low spin (singlet)	–12 449, 2.58	9.51	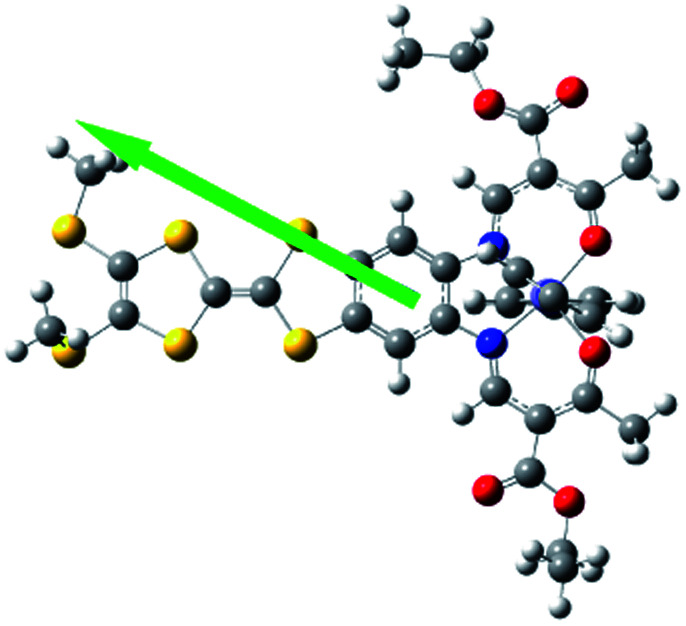	(0.00)	0.44

## Conclusion

In summary, by combining the redox-active TTF moiety which exhibits a relatively low redox potential, into a Schiff-base with a N_2_O_2_ square coordination, a one-dimensional Fe^II^ polymer was developed exhibiting unexpected coupling between spin-crossover and dielectricity enhancement. The reversible volume change of the Fe^II^ octahedron and bending of the TTF are coupled with the SCO. In light of the change in the TTF planarity with the oxidation state, we propose that it is potentially a concerted charge sharing between TTF and Fe centres. These results represent a new strategy towards the development of multifunctional molecular materials and provide impetus in the search for ferroelectricity coupled to spin-crossover.

## Funding sources

The authors declare no competing financial interests.

## Conflicts of interest

There are no conflicts to declare.

## Supplementary Material

Supplementary information

Crystal structure data
