# Supplementary material for: Enhanced dielectricity coupled to spin-crossover in a one-dimensional polymer iron(ii) incorporating tetrathiafulvalene
Source: Chem Sci. 2020 May 27;11(24):6229–35. doi: 10.1039/d0sc02388d (PMC7480181; doi:10.1039/d0sc02388d)
Supplement: Supplementary file 1 [file SC-011-D0SC02388D-s001.pdf]

Electronic Supplementary Material (ESI) for Chemical Science.
This journal is © The Royal Society of Chemistry 2020

Supporting Information

Enhanced Dielectricity Coupled to Spin-Crossover in a One-Dimensional Polymer Iron(II) Incorporating Tetrathiafulvalene

Ya-Ru Qiu,[‡a] Long Cui,[‡a] Pei-Yu Cai,[a] Fei Yu,[a,b] Mohamedally Kurmoo,*[a,c] Chanel F. Leong,[d] Deanna M. D'Alessandro,[d] Jing-Lin Zuo*[a]

[a] *State Key Laboratory of Coordination Chemistry, School of Chemistry and Chemical Engineering, Collaborative Innovation Center of Advanced Microstructures, Nanjing University, Nanjing, 210023, China.*

[b] *School of Chemistry and Materials Science, Nanjing University of Information Science and Technology, Nanjing, 210044, P. R. China.*

[c] *Institut de Chimie de Strasbourg, CNRS-UMR7177, Université de Strasbourg, 4 rue Blaise Pascal, Strasbourg 67000, France.*

[d] *School of Chemistry, The University of Sydney, Sydney, New South Wales, 2006, Australia.*

[‡] *These authors contributed equally to this work.*

**To whom correspondence should be addressed. Email: zuojl@nju.edu.cn; kurmoo@unistra.fr; kurmoo@nju.edu.cn*

Contents

1. Materials and Physical Measurements

2. Crystal Structures

Table S1. Selected bond lengths (Å) and angles (°) of the Fe^{II} coordination in **1** at different temperatures.

Figure S1. Views of the structures of **1** in its HS state at 350 K (left) and in its LS state at 123 K (right). H atoms are omitted for clarity. The S···S contacts between neighbouring TTF moieties are shown with cyan coloured sticks.

3. Density functional theory (DFT) calculations

4. Characterizations

Figure S2. Temperature dependence of $\chi_M T$ for **1** measured in 1 kOe from 2 to 400 K at a rate of 2 K min^{-1}.

Figure S3. The EPR spectra of **1** in the solid-state at 90 (red) and 300 K (black).

Figure S4. DSC for **1** recorded upon heating and cooling at rate of 15 (green), 10 (blue) and 5 (red) K min^{-1}.

Figure S5. Temperature dependence of real (ε') (a) and imaginary (ε'') (b) parts of dielectric function for **1** at different frequencies of 0.5, 1.0, 5.0, 10.0, 100.0, and 1000.0 kHz in both heating and cooling modes from 290 to 363 K at a rate of 20 K min^{-1}.

Figure S6. Solid-state CV for **1** over the potential range of -0.5 to 1.0 V vs. Fc/Fc^+ showing the first scan (red) and second scan (blue). CVs obtained at a scan rate of 100 μV/s in 0.1 M $[(n\text{-Bu})_4N]PF_6$ in CH_3CN supporting electrolyte.

Figure S7. Temperature dependence of the resistivity for **1** from 300 to 350 K at a rate of 1 K min^{-1}.

Figure S8. The TGA for **1** from 30-800 ℃ at a rate of 10 °C min^{-1} (The solvent has been removed prior to measurement).

Figure S9. The solid-state UV-vis absorption spectrum of **1** at room temperature.

Figure S10. The ^{1}H NMR spectrum for the ligand $\mathbf{H_2L}$ in $CDCl_3$.

Table S2. Some reported SCO compounds coupling between SCO behaviors and the dielectric properties.

5. References

1. Materials and Physical Measurements

All the reagents and solvents were commercially available and used as received. Dichloromethane, methanol and diethyl ether were all degassed prior to use. 2-(4,5-bis(methylthio)-1,3-dithiol-2-ylidene)benzo[*d*][1,3]dithiole-5,6-diamine was synthesized according to the literature.[S1] Elemental analyses (EA) for C, H and N were performed on a Perkin-Elmer 240C analyzer. Infrared (IR) spectra were recorded on a Vector 27 Bruker Spectrophotometer by transmission through KBr pellets containing the ground crystals in the range 4000-400 cm^{-1}. Thermogravimetric analyses (TGA) data were obtained on a STA 449C thermal analysis system from 30 to 800 °C at a heating rate of 10 °C min^{-1} under N_2 atmosphere. Differential scanning calorimetry (DSC) measurements were recorded using a METTLER-TOLEDO DSC1 differential scanning calorimeter with temperature sweep rates of 15, 10 and 5 K min^{-1}. Magnetization measurements for polycrystalline samples were performed using a Quantum Design SQUID vibrating sample magnetometer (VSM) in field of 1 kOe from 2 to 400 K at a rate of 2 K min^{-1}. Electron paramagnetic resonance (EPR) spectra were obtained with a Bruker EMX-10/12 X-band variable temperature apparatus at 90 and 300 K. ^{1}H-NMR spectra were measured on a Bruker AM 500 spectrometer. The solid-state UV-vis spectra for solid samples at room temperature were determined on a Shimadzu UV-3600 spectrophotometer. Cyclic voltammetry (CV) was performed using a BASi Epsilon Electrochemical Analyzer with ferrocene (Fc) as an internal reference. The solid sample of the material was mechanically immobilised on the surface of the working electrode. Measurements were conducted under an inert Ar atmosphere using a conventional three-electrode cell with a glassy carbon working electrode containing the immobilized solid, a Pt wire auxiliary electrode and an Ag/Ag^+ quasi reference electrode. A 0.1 M tetrabutylammonium hexafluorophosphate ($[(n\text{-Bu})_4N]PF_6$) in CH_3CN or DMF as supporting electrolyte was employed. Temperature dependence of the resistivity measurements for the samples using the two-probe method were performed on a Quantum Design SQUID physical property measurement system (PPMS) from 300 to 350 K at the rate of 1 K min^{-1}. The powdered samples were pressed at 1 Ton into pellets, which were connected by conductive silver adhesive.

Due to the very rapid loss of solvent of crystallization all reported measurements, except that for single-crystal diffraction at 123 K, were performed on fully desolvated samples.

2. Crystal Structures

Two sets of diffraction data were collected from two independent selected single-crystals of **1**. One crystal was used for the low temperature measurement at 123 K using a Bruker APEX DUO CCD diffractometer (Mo Kα). The other was used for the measurement at 350 K using a Bruker D8 Venture (Mo Kα). The raw frame data were integrated into SHELX-format reflection files and corrected for Lorentz and polarization effects using SAINT.[S2] Corrections

for incident and diffracted beam absorption effects were applied using SADABS.[S3] No change of diffraction intensity was evidence during data collection. The structures were solved and refined against F^2 by the full-matrix least-squares using the SHELXL-2018/3 program.[S4] The positions of the metal atoms and their first coordination spheres were located from direct method E-maps. All non-hydrogen atoms were refined with anisotropic thermal parameters, and hydrogen atoms of the organic ligands were calculated theoretically onto the specific atoms and refined isotropically with fixed thermal factors.The solvent contents in the crystal were removed from the data set using the SQUEEZE routine of PLATON and refined further using the data generated.[S5] In a unit cell, 42 electrons (158 Å^3) were squeezed and counted as 1 CH_2Cl_2. The solvent contents are not represented in the unit cell formula in the final formulas of **1-123K**, **1-350K**, **H_2L**. More details of the crystal data, data collection parameters, and refinement statistics for **1** and **H_2L** are given in Table 1. The comparison between C-C and C-S bond distances of the central TTF matrix for **1** and **H_2L** were depicted in Table 2. Selected bond lengths and bond angles of **1** were listed in Table S1 (Supporting Information). CCDC reference numbers: 1835730 (**1-123K**), 1835731 (**1-350K**) and 1942248 (**H_2L**). These data can be obtained free of charge from the Cambridge Crystallographic Data Centre via www.ccdc.cam.ac.uk/data_request/cif. All relevant data supporting the findings of this study are available from the corresponding authors on request.

Table S1. Selected bond lengths (Å) and angles (°) around Fe^{II} of **1** at different temperatures

	**1-123K**	**1-350K**
Fe(1)-O(2)	1.966(4)	1.988(9)
Fe(1)-O(1)	1.973(5)	2.000(8)
Fe(1)-N(1)	1.977(4)	2.086(10)
Fe(1)-N(2)	1.986(4)	2.071(8)
Fe(1)-N(3)	2.110(5)	2.223(11)
Fe(1)-N(4)	2.090(5)	2.171(11)
O(1)-Fe(1)-O(2)	99.10(18)	110.1(3)
N(2)-Fe(1)-N(1)	82.23(16)	79.7(4)
O(1)-Fe(1)-N(3)	90.24(18)	87.5(3)
O(2)-Fe(1)-N(3)	88.62(17)	88.0(4)
N(1)-Fe(1)-N(3)	94.20(17)	90.5(4)
N(1)-Fe(1)-N(4)	90.16(17)	92.1(4)
N(2)-Fe(1)-N(4)	92.24(17)	92.3(4)
N(3)-Fe(1)-N(4)	175.42(19)	176.1(4)

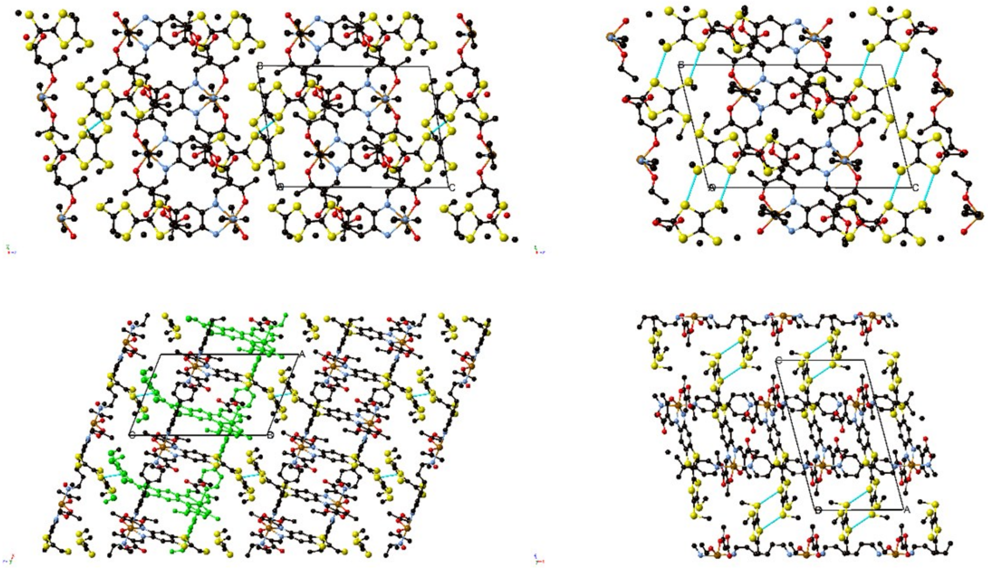

Figure S1. Views of the structures of **1** in its HS state at 350 K (left) and in its LS state at 123 K (right). H atoms are omitted for clarity. The S···S contacts between neighboring TTF moieties are shown with cyan coloured sticks.

3. Density functional theory (DFT) calculations

Single-point energy calculations for the X-ray determined structures at singlet (LS) and quintet (HS) spin states were carried out by using density functional theory (DFT) methods. The spin unrestricted strategy was applied for HS state. The Gaussian 09 program package[S6] was utilized. The b3lyp functional together with 6-31G** basis set for all atoms were adopted

(Table 3). DFT calculations were carried out in the singlet (LS) and quintet (HS) spin states using geometries obtained in the X-ray crystal structures of **1**.

However, it is hard to obtain accurate value of the electric dipole via DFT calculations for such a one-dimensional compound. In this manuscript, the DFT calculation was utilized to support the change tendency in charge distribution and dielectric properties accompanying Fe^{II} sites and the TTF building blocks. In addition, the 5.14 D difference between the low spin and high spin phases in electric dipole is much smaller than that of similar Fe^{II} compounds showing charge transfer (3.97 D or 16.7 D).[S7,S8]

4. Characterisations

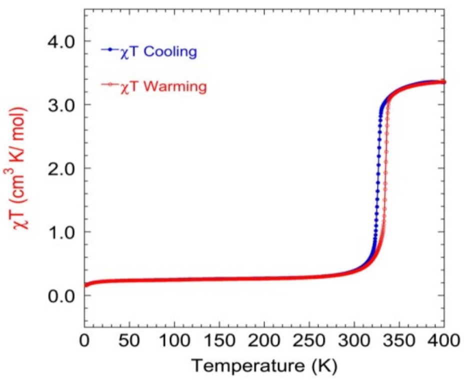

Figure S2. Temperature dependence of $\chi_M T$ for **1** measured in 1 kOe from 2 to 400 K at a rate of 2 K min^{-1}.

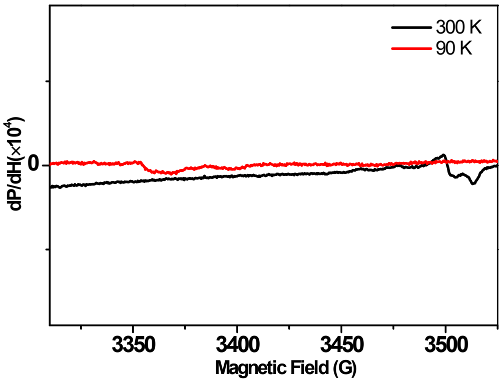

Figure S3. The EPR spectra of solid-state samples of **1** at 90 K (red line) and 300 K (black line).

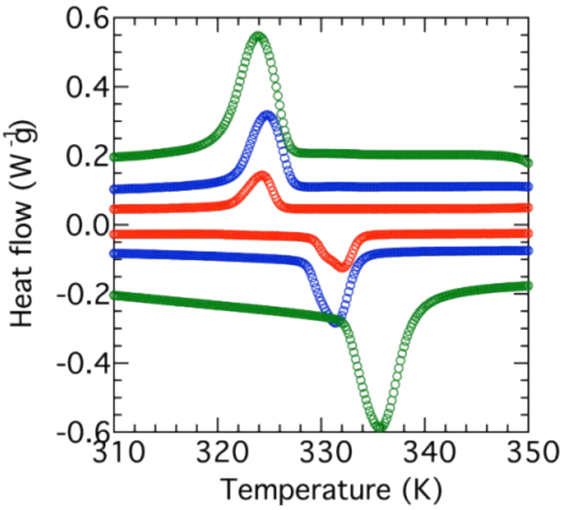

Figure S4. DSC for **1** recorded upon heating and cooling modes at rate of 15 (green), 10 (blue) and 5 (red) K min^{-1}.

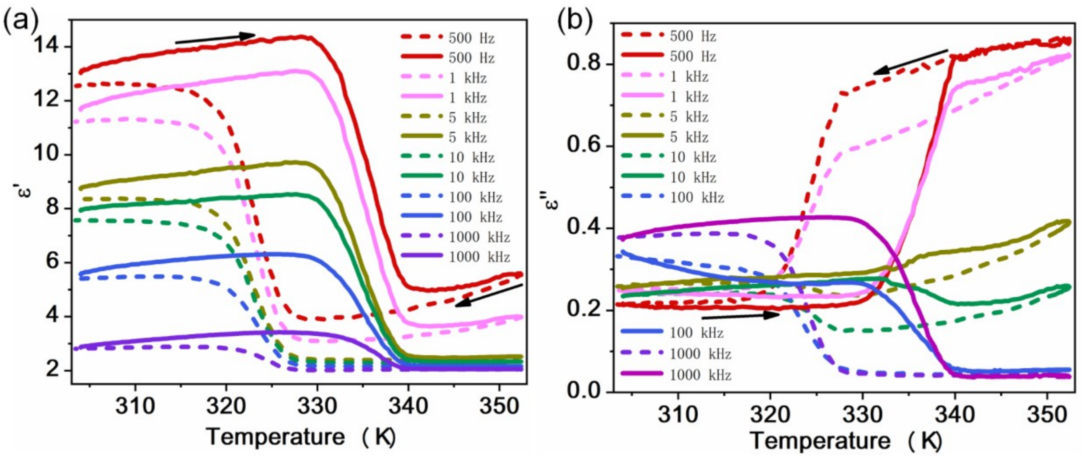

Figure S5. Temperature dependence of real (ε') (a) and imaginary (ε'') (b) parts of dielectric function for **1** at different frequencies of 0.5, 1.0, 5.0, 10.0, 100.0, and 1000.0 kHz in both heating and cooling modes from 290 to 363 K at a rate of 20 K min^{-1}.

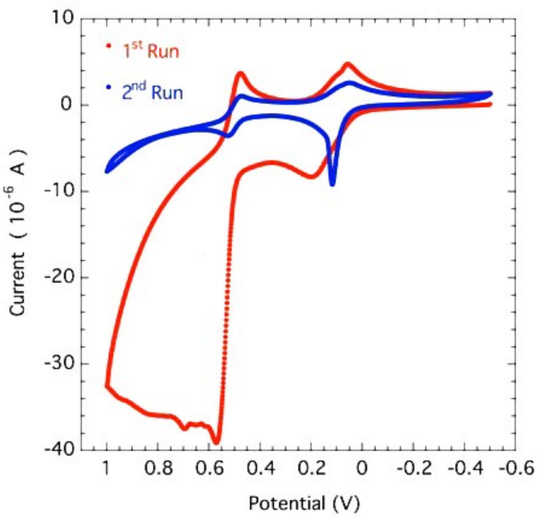

Figure S6. CV for a solid sample of **1** over the potential range of -0.5 to 1.0 V *vs.* Fc/Fc$^+$ showing the first scan (red) and second scan (blue). CVs obtained at a scan rate of 100 μV/s in 0.1 M [(n-Bu)$_4$N]PF$_6$ in CH$_3$CN supporting electrolyte.

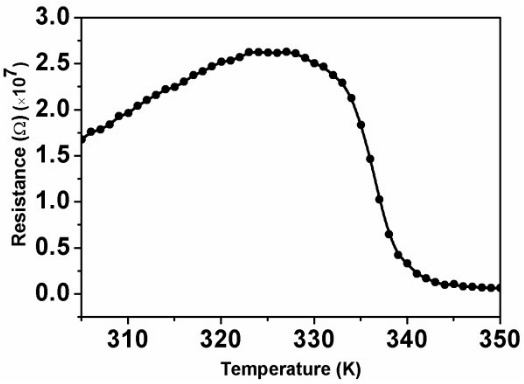

Figure S7. Temperature dependence of the resistivity for **1** from 300 to 350 K at rate of 1 K min^{-1}.

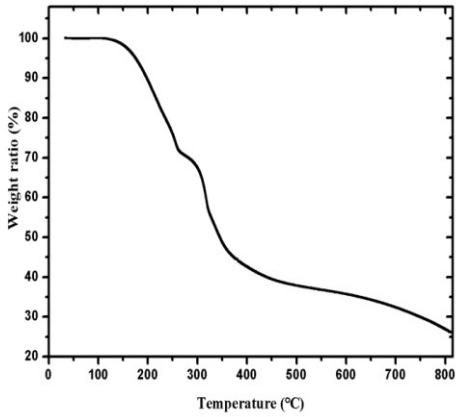

Figure S8. The TGA for **1** from 30-800 ℃ at rate of 10 °C min^{-1} (The solvent has been removed prior to measurement).

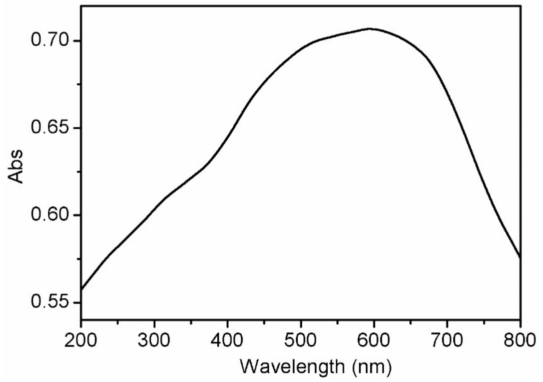

Figure S9. The solid-state UV-vis absorption spectrum of **1** at room temperature.

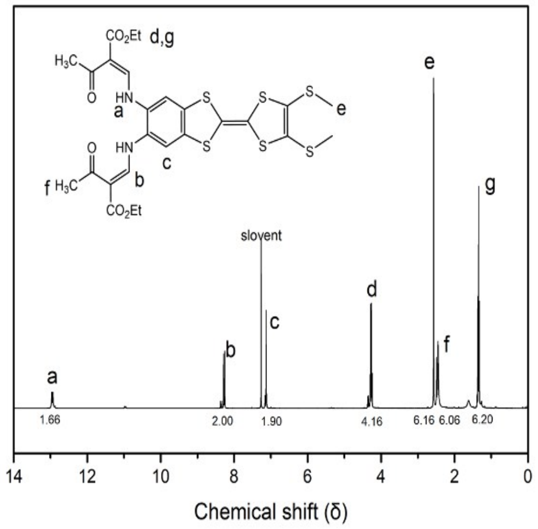

Figure S10. The ^{1}H NMR spectrum for ligand $\mathbf{H_2L}$ in $CDCl_3$.

Table S2. Some reported SCO compounds coupling between SCO behaviors and the dielectric properties.

Molecular Structure	$T_{1/2}\uparrow$ (K)	$T_{1/2}\downarrow$ (K)	Dielectric constant	References
$Fe_{0.8}Ni_{0.2}(btr)_2(NCS)_2\cdot H_2O$	146	126	3.7	15a
$[Fe(NH_2trz)_3](NO_3)_2$	337	287	4.5	15a
Fe[5NO$_2$-sal-N(1,4,7,10)]	145 $T_{1/2}(1)$; 176 $T_{1/2}(2)$	135 $T_{1/2}(1)$; 172 $T_{1/2}(2)$	5.0	15a
$\{[(Tp)Fe^{III}(CN)_3]_2Fe^{II}(azp)\cdot 4H_2O$	171	169	5.3	15b
[Fe{(R)-L}(bpz)$_2$]	189	187	3.5	15c
$[Fe_2L_2(\mu\text{-}L)_3(NCSe)_4]\cdot 2DMF\cdot 2H_2O$	164	164	5.0	15d
[Mn(taa)]	48	48	7.0	15e

btr = [4,4'-bis-1,2,4-triazole]$_2$; NH_2trz = 4-amino-1,2,4-1H-triazole; sal = salicylaldhyde.
Tp = hydrotris(pyrazolyl)borate); azp = 4,4'-azopyridine.

L = (R)/(S)-4,5-pinenepyridyl-2-pyrazine; bpz = bis(1-pyrazolyl)borohydride.
L = 1-naphthylimino-1,2,4-triazole.
H_3taa = tris(1-(2-azolyl)-2-azabuten-4-yl)amine.

5. References.

S1 (a) C. Jia, S.-X. Liu, C. Tanner, C. Leiggener, A. Neels, L. Sanguinet, E. Levillain, S. Leutwyler, A. Hauser and S. Decurtins, *Chem. Eur. J.* 2007, **13**, 3804-3812; (b) X. Guégano, A. L. Kanibolotsky, C. Blum, S. F. L. Mertens, S.-X. Liu, A. Neels, H. Hagemann, P. J. Skabara, S. Leutwyler, T. Wandlowski, A. Hauser and S. Decurtins, *Chem. Eur. J.* 2009, **15**, 63-66; (c) J.-C. Wu, S.-X. Liu, T. D. Keene, A. Neels, V. Mereacre and A. K. Powell and S. Decurtins, *Inorg. Chem.* 2008, **47**, 3452-3459.

S2 *SAINT-Plus, version 6.02*; Bruker Analytical X-ray System, Madison, WI, 1999.

S3 Sheldrick, G. M. SADABS, *an empirical absorption correction program*; Bruker Analytical X-ray Systems, Madison, WI, 1996.

S4 Sheldrick, G. M. Crystal structure refinement with SHELXL. *Acta Crystallogr., Sect. C: Struct. Chem.* 2015, **71**, 3-8.

S5 Spek, A. L. *J. Appl. Crystallogr.* Single-crystal structure validation with the program PLATON. **2003**, *36*, 7.

S6 Gaussian 09, Revision A.01, M. J. Frisch, G. W. Trucks, H. B. Schlegel, G. E. Scuseria, M. A. Robb, J. R. Cheeseman, G. Scalmani, V. Barone, B. Mennucci, G. A. Petersson, H. Nakatsuji, M. Caricato, X. Li, H. P. Hratchian, A. F. Izmaylov, J. Bloino, G. Zheng, J. L. Sonnenberg, M. Hada, M. Ehara, K. Toyota, R. Fukuda, J. Hasegawa, M. Ishida, T. Nakajima, Y. Honda, O. Kitao, H. Nakai, T. Vreven, J. A. Montgomery, Jr., J. E. Peralta, F. Ogliaro, M. Bearpark, J. J. He yd, E. Brothers, K. N. Kudin, V. N. Staroverov, R. Kobayashi, J. Normand, K. Raghavachari, A. Rendell, J. C. Burant, S. S. Iyengar, J. Tomasi, M. Cossi, N. Rega, J. M. Millam, M. Klene, J. E. Knox, J. B. Cross, V. Bakken, C. Adamo, J. Jaramillo, R. Gomperts, R. E. Stratmann, O. Yazyev, A. J. Austin, R. Cammi, C. Pomelli, J. W. Ochterski, R. L. Martin, K. Morokuma, V. G. Zakrzewski, G. A. Voth, P. Salvador, J. J. Dannenberg, S. Dapprich, A. D. Daniels, Ö. Farkas, J. B. Foresman, J. V. Ortiz, J. Cioslowski, D. J. Fox, Gaussian, Inc., Wallingford CT, 2009.

S7 H. Zheng, Y.-S. Meng, G.-L. Zhou, C.-Y. Duan, O. Sato, S. Hayami, Yi Luo and T. Liu, *Angew. Chem. Int. Ed.* 2018, **57**, 8468-8472.

S8 J.-X. Hu, L. Luo, X.-J. Lv, L. Liu, Q. Liu, Y.-K. Yang, C.-Y. Duan, Y. Luo and T. Liu, *Angew. Chem. Int. Ed.* 2017, **56**, 7663-7668.
